# Evolutionary Debunking Arguments

**DOI:** 10.1111/j.1468-0068.2010.00770.x

**Published:** 2011-03

**Authors:** Guy Kahane

**Affiliations:** University of Oxford

## Abstract

Evolutionary debunking arguments (*EDA*s) are arguments that appeal to the evolutionary origins of evaluative beliefs to undermine their justification. This paper aims to clarify the premises and presuppositions of EDAs—a form of argument that is increasingly put to use in normative ethics. I argue that such arguments face serious obstacles. It is often overlooked, for example, that they presuppose the truth of metaethical objectivism. More importantly, even if objectivism is assumed, the use of EDAs in normative ethics is incompatible with a parallel and more sweeping global evolutionary debunking argument that has been discussed in recent metaethics. After examining several ways of responding to this global debunking argument, I end by arguing that even if we could resist it, this would still not rehabilitate the current targeted use of EDAs in normative ethics given that, if EDAs work at all, they will in any case lead to a truly radical revision of our evaluative outlook.

The worry that the theory of evolution is incompatible with morality and value is as old as the theory itself. This worry can take various forms, but the basic idea is familiar. Some people think that if we are the products of blind natural selection, then morality and value are merely reflections of our subjective attitudes, and that in that case everything is permitted and nothing matters. But since that would be absolutely awful, the theory of evolution couldn't be true.

There is more than one thing wrong with this argument. It starts with a problematic step from a naturalist claim about the origins of morality to a subjectivist or similar anti-objectivist view of value and morality. But this causal claim, and naturalism in general, are compatible with many variants of objectivism.^1^ And in any case, anti-objectivism is a metaethical view, a view about the *semantics* of evaluative statements, and naturalism or the evolutionary origins of morality do not by themselves support such a semantic view. Furthermore, even if naturalism did imply anti-objectivism, it is confused to think that anti-objectivism implies evaluative nihilism. After all, anti-objectivist metaethical views are attempts to give an account of existing evaluative discourse, and as such, if successful, should leave our first-order evaluative beliefs exactly as they are. Finally, even if our evolutionary origins did imply nihilism, this wouldn't be a good reason to reject the theory of evolution. It is not rational to reject the premises of an argument just because we are disturbed by its conclusion. And if the upshot of the argument was not only moral nihilism but general evaluative nihilism, then what is it exactly we're supposed to be afraid of? If nothing matters then it doesn't matter that nothing matters.

Such anxiety about evolution has hardly disappeared but when Ronald Dworkin writes that “the widespread assumption that a successful Darwinian explanation of moral concern … would have sceptical implications” is “plainly mistaken,”^2^ he is reporting the view of most philosophers. There is nevertheless, I believe, a persistent sense of unease about our evolutionary origins even in many who reject this bad argument, a felt tension between our immersed evaluative standpoint and the story evolutionary theory tells about its origins. A tension, for example, between an unreserved appreciation of someone's beauty and the recognition that our responses to physical beauty are merely evolved dispositions whose function is to help us track indicators of fertility, health and freedom from parasites in potential mates—all in the service of spreading our genes.

Bad arguments often have a psychological afterlife, exerting a hidden influence even after they have been consciously disavowed. Our unease might be due to such influence. But there are other ways in which our evaluative beliefs might be in tension with our evolutionary origins. The evolutionary argument I shall be considering in this paper does not try to establish any metaphysical claim about the existence of values. It is rather an *epistemic* argument that claims that the evolutionary origins of certain evaluative beliefs undermine their justification. I'll call arguments that take this form *evolutionary debunking arguments* (EDAs).

Whether in implicit or explicit form, EDAs are increasingly being put to use in recent normative ethics. I shall argue that EDAs are merely instances of a familiar form of argument that is commonly used both in evaluative and non-evaluative contexts. I shall sketch a general schema for such arguments in section I, and in section II I shall show how this schema can be applied to beliefs that have their source in evaluative dispositions selected by evolution, using several examples from recent normative ethics as illustrations. In these examples, EDAs are used in a targeted way in aid of substantive evaluative claims. However, as will emerge in section III, a parallel form of argument has been used in recent metaethics to defend a far more sweeping conclusion that is not compatible with this targeted use. In section IV, I shall consider several ways of resolving the tension. One thing that will emerge is that although the common view that competing metaethical views ought to be compatible with our first-order evaluative beliefs is correct so far as it goes, the relation between metaethics and normative ethics is less straightforward than often assumed.

## I Debunking Arguments

The idea that the aetiology of a belief can undermine its epistemic standing is far from new. It actually needs rehabilitation after decades of misuse in certain areas of the humanities and social sciences. It is common for authors in these areas to slide from some surprising hypothesis about the causal sources of some view, to treating that hypothesis as firmly established, and, then, to concluding that this view has thereby been thoroughly discredited.^3^

Since this form of argument has often been used by theorists of a Marxist bent, it seems appropriate to start with an example drawn from recent speculations about Karl Marx himself—the suggestion that Marx's views on alienation have their source in the fact that he suffered from *hidradenitis suppurativa*, an agonizing skin disease said to cause self-loathing.^4^ It would be absurd, however, to think that if this medical hypothesis is correct, then this would in itself cast any doubt on Marx's substantive claims. To reject Marx's claims about alienation, we need to find flaws in the *reasons* he gave for them. It seems irrelevant whether these claims were causally shaped by ruthless ambition, a skin condition, or an unresolved Oedipus complex. To think otherwise, it would seem, would be to commit the genetic fallacy, to confuse causes and reasons.

As a general principle, it is true that when we consider a proposition someone has put forward, we should focus on the balance of reasons in its favour, not on our adversary's biography. But this point is compatible with the narrower and unremarkable claim that, when certain conditions are met, the causal origins of a belief can reduce or even remove its justification. Obviously, the mere fact that there is a causal explanation of a belief does nothing to affect its justification. All beliefs have a causal explanation. But if someone decided whether or not to believe that p by flipping a coin, her belief would surely be unjustified; there is simply no connection whatsoever between this means of forming a belief and the truth. What matters here is not whether a belief was shaped by a process that is literally random but whether it was shaped by a process that *tracks* the truth. I'll call processes that are not truth-tracking ‘off track’. Now the mere fact that a belief was formed in such a way would not, on some views of justification, undermine justification in it so long as the believer is unaware of this off track influence. But on any plausible view of justification she would not be justified in holding on to the belief once this fact was brought to her attention. The second-order belief that a certain belief was formed by an off track process offers an *undermining defeater* for that belief.^5^

Most off track influences on belief are far from random. One large class of such processes are various types of motivated cognition—the distorting influence on belief exerted by self-interest, vanity, or pessimism. Think, for example, of the doting mother who believes that whatever her son does is admirable—and would think so whether the son did one thing or its very opposite. Everyone agrees that both evaluative and non-evaluative beliefs can be biased in these ways. When we criticize such biasing influences on belief, we are putting forward *psychological* debunking arguments.

Debunking arguments are arguments that show the causal origins of a belief to be an undermining defeater. The basic structure of such arguments is simple:

*Causal premise.* S's belief that p is explained by X.

*Epistemic premise*. X is an off-track process.

Therefore

S's belief that p is unjustified.^6^

There is much that could be said to elucidate these premises. Here I shall simply assume an intuitive understanding of the epistemic premise (such an understanding doesn't require a positive account of truth-tracking processes), but I shall say a bit more about the causal premise. It is not enough if a causal explanation cites factors that are off track. The full causal explanation of any belief will inevitably cite such factors. Perhaps if Einstein had greater musical talent, he would not have developed an interest in physics and not come to believe the theory of relativity. But the truth of this counterfactual does nothing to affect the standing of his belief in the theory. The role of the off track process in the explanation must be such that it leaves no space for the contribution of processes that would, in this context, track the truth.

Suppose that the medical hypothesis about Marx meets this condition. Perhaps his skin condition does explain his views about alienation in this ambitious sense—that it didn't, for example, merely serve to draw his attention to something he had independent reason to believe. Still, even if the skin condition explained the *formation* of his belief in the relevant sense, it might be that this motivated him to seek good reasons for this belief, and that these good reasons are what sustained his belief over time. So our understanding of the causal premise also needs to rule out what we might call *post hoc justification*—to be distinguished from mere *post hoc rationalization*, where it is not only that a person is motivated to justify some belief, but the (claimed) justification is itself motivated—that is, shaped by off track influences.

It might be objected that even if the reasons Marx gave for his beliefs were themselves shaped by influences that are off track, these might still happen to be good reasons, even if they were not truly the (explanatory epistemic) reasons for his beliefs. We ought to engage these reasons directly. It is only if we can independently show them to be plainly bad reasons that the subsidiary task of explaining how anyone would come to endorse them might be of interest.

This objection fails to distinguish the question whether someone's belief *is* justified from the question whether it *could* be justified, possibly even in light of reasons that are already at hand. But even the hold of this distinction reaches only as far as reasons can reach. At some point reasons run out, and in evaluative matters that point is often reached. Some evaluative beliefs are supported by other, more basic ones, but ultimately we can't help but appeal to intuitions, whether to directly support some evaluative belief or as input to reflective equilibrium. Debunking explanations of such intuitions can leave a belief lacking both actual and alternative support.

To be sure, beliefs supported by intuition might nevertheless also be supportable by independent reasons. For example, a deontological intuition about a given case might also be supported by the Categorical Imperative. In some cases it is easy to rule out this possibility. Beliefs about intrinsic value seem to be based simply on reflection on the intrinsic properties of things. It is hard to see how the belief that pain is intrinsically bad could be supported by some independent evaluative principle. But appeal to the Categorical Imperative would also be ruled out if our belief in it was itself the product of a process of reflective equilibrium that took the debunked intuition as its input. Indeed, even if the Categorical Imperative was claimed to be derived from pure reason, this would still not help if the debunker could show that belief in it was itself causally driven by debunked intuitions, whether or not these are claimed to play any justificatory role.

In any case, if an evaluative belief was exclusively based on intuition, then, if this intuition is debunked, it would normally mean little that an alternative justification for this belief *might* be available. Normally we seek further justification for a view *because* we have some reason to think it's true. But if we conclude that the intuition that supports the belief has no epistemic force, why on earth should we look for an alternative justification?

### Debunking arguments and evaluative practice

Debunking arguments can target all types of belief—the attempted debunking of certain intuitions has played a central role in debates about consciousness—and this includes evaluative belief, as is familiar from everyday evaluative practice. Roger Crisp, for example, writes that for an evaluative intuition to be justified,

[it] should be neither a gut reaction nor an instinct nor something accepted purely on the basis of external authority, but a belief which to ‘careful observation’ presents itself as a dictate of reason …

And he adds that much of the reflection that validates the intuition will concern its origin. One should ask,

Is [the intuition] a product of my upbringing? Am I adopting it merely on the basis of societal or other authority? Does it rest on my subjective likes and dislikes?^7^

As Crisp describes evaluative reflection, much of it is concerned with ensuring that our intuitions aren't shaped by off track influences.

What work can debunking arguments do in normative ethics? It is notoriously hard to resolve disagreements about the supposed intrinsic value or moral significance of certain considerations—to resolve differences in intuition. And we saw that a belief's aetiology makes most difference for justification precisely in such cases, when reasons have run out. Debunking arguments thus offer one powerful way of moving such disagreements forward.

It's important not to overestimate what debunking arguments can achieve. All they can show is that someone is not justified in *believing* that p; although they can show the falsity of second-order beliefs about justification, on their own they do nothing to show that p itself is *false*. Debunking arguments are purely negative. If we show that belief in p is unjustified, it does not follow that belief in not-p is justified (the debunking of the belief in p can at most raise the relative justification of not-p). And notice that debunking arguments can apply to *both* sides of a dispute: there might be debunking explanations of beliefs in p *and* of beliefs in not-p.

### Historical debunking

I've already mentioned the widespread use of *psychological* debunking arguments. Another familiar form of such arguments is *historical*, most famously as used by Marx and Nietzsche.^8^ Nietzsche thought that ‘historical refutation’ is the ‘definitive refutation’.^9^ He somewhere remarks, for example, that

How [the belief in God]*originated* can at the present stage of comparative ethnology no longer admit of doubt, and with the insight into that origin the belief *falls away*.^10^

And he famously offered a debunking genealogy of the origins of Judeo-Christian ‘slave morality’ in the resentment of the weak towards the strong.^11^

Utilitarians such as Peter Singer also often appeal to such historical explanations. Singer writes, for example, that

On abortion, suicide, and voluntary euthanasia … we may think as we do because we have grown up in a society that was, for two thousand years, dominated by the Christian religion.^12^

The idea is that we should treat as suspect moral beliefs whose origins include off track influences such as the residue of arbitrary cultural conventions and mistaken supernatural beliefs, or the self-interested motivation of one group to subdue another.

It's easy to find other examples of debunking arguments in recent normative ethics that take a broadly historical form. For example, Mulgan points out that we cannot take at face value the philosophical consensus that Nozick's example of the experience machine is a decisive objection to hedonism because many undergraduates do not find it convincing and it is not surprising that few of *these* make it to graduate school let alone into professional philosophy.^13^ And Crisp suggests that we value accomplishment independently of pleasure only because early societies that valued accomplishment did better than those that didn't.^14^

## II Evolutionary Debunking Arguments

A long established use of historical debunking arguments assumes that our natural doxastic dispositions are indicative of truth. Thomas Reid, for example, claimed that our confidence in a belief should be greater if we formed it naturally, “before we could reason, and before we could learn it by instruction.”^15^ If we are the designed products of God, then it does seem rational for us to rely on our natural doxastic dispositions given that these were implanted in us by an omniscient and omnibenevolent being.^16^

We can no longer trust our nature in this way. Sidgwick already wrote that “[i]t is hardly necessary at the present day to point out how entirely [Reid's] assumption lacks scientific foundation,” though he was still reluctant to endorse to opposite, negative claim.^17^ In recent discussion we find no such reluctance. Michael Huemer, for example, writes that “[e]volution may have endowed us with biases that affect our moral judgements. Sociobiology can help us identify these biases and so correct for them, thereby improving moral cognition.”^18^ It is now common to think of nature as a *distorting* influence on our evaluative beliefs (without necessarily implying that culture is any better—that it plays any kind of correcting role). And implicit or explicit debunking arguments that rely on this assumption are fairly widely used in contemporary normative ethics. Here are just three examples.

The first is Derek Parfit's discussion of our attitudes to time. Parfit argues that it is irrational to prefer great agony in the further future to weaker pain sooner, or to prefer pain in the past to lesser pain in the future. And he points out that this common pattern of attitudes has an obvious evolutionary explanation. Parfit, however, thinks that the epistemic implications of this explanation are limited. He writes that

if some attitude has an evolutionary explanation, this fact is neutral. It neither supports nor undermines the claim that this attitude is justified. But there is one exception. It may be claimed that, since we all have this attitude, this is a ground for thinking it justified. *This* claim is undermined by the evolutionary explanation. Since there is this explanation, we would all have this attitude even if it was not justified; so the fact that we have this attitude cannot be a reason for thinking it justified. Whether it is justified is an open question, waiting to be answered.^19^

This passage makes it seems as if evolutionary explanations only undermine the epistemic significance of wide agreement. But this is too weak. A debunking argument does not show that an evaluative attitude is unjustified, but it *can* show that the *belief* that this attitude is justified is unjustified. And although it sometime remains an open question whether this belief can be justified in some other way, in other cases, when it's doubtful that the belief can be supported by anything other than intuition, it will not be an open question whether the proposition might still be justified.

Crisp has recently argued that, in the face of pervasive disagreement on evaluative matters, we should have greatest confidence in the rationality of self-interest—a normative principle supported by an intuition that Crisp thinks uniquely passes the tests cited above.^20^ But there is an obvious evolutionary explanation of self-interested concern. As Stuart Rachels and Torin Alter point out

Evolution favors the selfish; animals that care more about themselves will, on average, reproduce more than animals that do not … evolution ensures that we care more about our own pain and death than that of others. Because of this, we are encouraged to think we have a special reason to care about our own death and pain.

But this evolutionary point

calls into question any such intuitive appeal, since the evolutionary process aims at fitness, not truth, wisdom, or rational attitudes. So our opponents cannot assume that the basic intuition captures some philosophical insight … Evolution and psychology can do all the explanatory work; no appeal to insight is needed.^21^

Finally, Joshua Greene and Peter Singer use debunking arguments against deontological intuitions. In a discussion of common deontological intuitions about the supposed moral difference between diverting a trolley so that five persons are saved at the cost of one's death and directly pushing one person onto the trolley's track to prevent five from dying, Greene suggests that these intuitions are merely due to the fact that ‘up close and personal’ violence was around for a long time and a powerful negative response to such violence was selected by evolution, whereas indirect ways of killing others are relatively recent and therefore arouse no such innate response.^22^ Remarking on this suggestion, Singer asks,

[w]hat is the moral salience of the fact that I have killed someone in a way that was possible a million years ago, rather than in a way that became possible only two hundred years ago? I would answer: none.^23^

Greene and Singer take these considerations to have momentous consequences for the dispute between deontologists and utilitarians and quite generally for the practice of normative ethics. Greene thinks that such explanations show that deontology is merely “a kind of moral confabulation”^24^ while Singer thinks that they give us powerful new grounds for rejecting the pervasive practice of appealing to intuition in normative ethics.^25^

These three arguments can be set out as instances of the general schema for debunking arguments:

*Causal premise.* We believe that p, an evaluative proposition, because we have an intuition that p, and there is an evolutionary explanation of our intuition that p.

*Epistemic premise.* Evolution is not a truth-tracking process with respect to evaluative truth.

Therefore

We are not justified in believing that p.

I don't have much to say about the causal premise except to emphasize that it makes an extremely ambitious empirical claim. Just as the historical explanations suggested by Nietzsche and Singer are at best sketchy speculations,^26^ the evolutionary explanations in the above examples, as plausible as they may sound, are a long way from even beginning to fill out the empirical details needed to fully secure this premise. Still, for the argument to go through it might be enough if enough is said to make the truth of this premise significantly more plausible than the converse claim that the belief was formed by processes that are truth tracking.

It's important to see that it does not matter here whether any *particular* evolutionary explanation is true. What matters is that *some* such story is likely to be true. Moreover, the evolutionary explanations given in the above examples can create the misleading (and apparently common) impression that that EDAs must presuppose the general cogency of evolutionary psychology or a strong adaptationist understanding of evolution.^27^ But this is a mistake. If some evaluative disposition is explained not by adaptation but by the even *more* random evolutionary mechanisms of genetic drift or exaptation, this would make things *worse*, not better, with respect to truth tracking. It would make the process even *more* similar to flipping a coin. So it makes little sense to try to resist EDAs by showing that some evaluative disposition has its origins in a non-adaptive evolutionary process. This would be a bit like a libertarian incompatibilist who thought that we can defend free will by showing that our choices are completely random.

What does seem to me true is that EDAs commit us to claims about innateness—not necessarily of the evaluative beliefs themselves, but of strong dispositions that push people in their direction.^28^ Indeed it seems that a far simpler form of EDA than in the examples given would be to establish that a certain evaluative disposition is innate. If none of the processes that might explain our innate evaluative dispositions is plausibly truth-tracking, it should make no difference whether we have an adaptive explanation for this disposition.

The epistemic premise claims that natural selection is a process that does not track evaluative truth. I am not going to argue for this claim, which has been defended at length by others.^29^ But note that this is a claim only about evaluative truth, not a claim about truth in general. I'll be making the widely held assumption that evolutionary explanations of our perceptual capacities *do* need to cite the reliability of these capacities in tracking empirical truths.^30^

### *An overlooked metaethical commitment*?

There is an important presupposition of EDAs which is typically overlooked by debunkers in normative ethics. Such arguments appear to presuppose the truth of *objectivism*. After all, anti-objectivist views claim that our ultimate evaluative concerns are the source of values; they are not themselves answerable to any independent evaluative facts. But if there is no attitude-independent truth for our attitudes to track, how could it make sense to worry whether these attitudes have their distal origins in a truth-tracking process? The epistemic premise of EDAs therefore seems to assume the truth of the following *metaethical* thesis:

*Metaethical assumption.* Objectivism gives the correct account of evaluative concepts and properties.^31^

I shall return to this assumption below. At present, let me just draw attention to some odd implications it would have. It would mean that your metaethical view can make a dramatic difference to the range of substantive evaluative views you can justifiably believe. It would mean, for example, that simply by changing your metaethical view from anti-objectivism to objectivism you might also be rationally required to change your substantive views about time or self-interest, or to move from a Kantian ethics to a utilitarian one. And this means that the way EDAs are currently deployed in normative ethics is misleading. These arguments have force *only* against objectivist opponents. An anti-utilitarian such as Bernard Williams who rejects objectivism has no reason to be impressed by the debunking arguments of Greene and Singer.^32^

### *Where does the debunking stop*?

If you cite an off track causal influence on an interlocutor's belief that p in order to increase support for your view that not-p, you should, at the minimum, first rule out that your own belief was shaped by this or a similar influence. When Singer appeals to an historical explanation to debunk certain moral beliefs, we can wonder whether he goes far enough. After all, Nietzsche *also* claims that the Judeo-Christian heritage of Western thought has seriously distorted our moral outlook. But Nietzsche would see Singer's utilitarianism as itself a paradigmatic example of such a distorted residue of Judeo-Christian morality. There is the danger, then, that the reach of a debunking argument would extend further than intended. Writing about altruism, Greene asks us to suppose that

the *only* reason that faraway children fail to push our emotional buttons is that we evolved in an environment in which it was impossible to interact with faraway individuals. Could we then stand by our commonsense intuitions? Can we, in good conscience, say, “I live a life of luxury while ignoring the desperate needs of people far away because I, through an accident of human evolution, am emotionally insensitive to their plight. Nevertheless, my failure to relieve their suffering, when I could easily do otherwise, is perfectly justified.” … I find this combination of assertions uncomfortable.^33^

Even if we find this combination uncomfortable, this would not necessarily support utilitarianism. Rather, two possibilities will be left open: we should either generalise altruism to cover all cases, or not care at all. And evolutionary considerations should support the latter possibility, given that accidents of human evolution explain not only our different beliefs about helping nearby and faraway individuals, but also the very belief that we ought to help others at all. And this means that we can easily mount a Nietzschean counter argument from the standpoint of rational egoism, asking whether we can rationally believe, “I must sacrifice my own welfare for the sake of others because I, through an accident of human evolution, am emotionally sensitive to their plight. Nevertheless, this sacrifice, when I could easily do otherwise, is perfectly justified.” Isn't this combination of assertions similarly uncomfortable?

If this ‘Nietzschean’ argument was successful, it would seem to justify the old religious worry that if naturalism is true then ‘everything is permitted’. But of course the debunking doesn't stop here, as the Rachels and Alter argument shows. Our self-interested evaluative dispositions are, surely, at least as susceptible to evolutionary explanation, as are our evaluative beliefs about pleasure, pain and death, and one may wonder whether the EDA can be stopped from covering the whole of the evaluative—from supporting, not utilitarianism or even rational egoism, but global evaluative scepticism. (Is utilitarianism merely half-hearted scepticism?)

## III The Global Debunking Argument

There is in fact an evolutionary metaethical argument that claims to establish precisely that. Richard Joyce's version of this argument starts with the empirical premise that “moral judgments have a certain kind of genealogy,” a genealogy that has at its core an account of “the evolution of the moral sense in the hominid lineage.”^34^ The claim is that our moral beliefs have been shaped by our contingent evolutionary history. As Darwin pointed out, if things had gone differently and humans have evolved to be more similar to bees, “[u]nmarried females would, like the worker-bees, think it a sacred duty to kill their brothers, and mothers would strive to kill their fertile daughters; and on one would think of interfering”.^35^ The problem is not that there is such a genealogical story to be told but that the story that can be told about the origins of our moral beliefs is one which “nowhere presupposes that the beliefs in question are true,”^36^ as shown by the point that

an acquaintance with the contemporary literature on the evolution of the human moral sense will reveal no background assumption that any actual moral rightness or wrongness existed in the ancestral environment.

This is hardly surprising given that natural selection is “a process for which practical success rather than accuracy is the summum bonum.”^37^ What Joyce claims follows from this is that

our moral beliefs are products of a process that is entirely independent of their truth, which forces the recognition that we have no grounds one way or the other for maintaining these beliefs

or, in other words, moral scepticism.^38^

Sharon Street has independently developed a similar argument, set out as a dilemma for objectivists.^39^ Street's core argument can also be laid out as two premises leading to a sceptical conclusion. Like Joyce, she starts with the premise that natural selection has been an enormous factor in shaping the content of the evaluative beliefs of humans, with the consequence that our evaluative beliefs are “thoroughly saturated with evolutionary influence.”^40^ Natural selection must have had such an influence given that different evaluative tendencies can have extremely different effects on a creature's chances of survival and reproduction: judging life to be valuable tends to increase reproductive success, cherishing death doesn't.

Given this empirical fact, objectivists face a dilemma: they must either claim that natural selection tracks objective value, an implausible empirical claim, or admit that there is no relation between natural selection and objective value—admit, that is, that evolution is ‘off track’, that natural selection is

a purely distorting influence on our evaluative judgments, having pushed us in evaluative directions that have nothing whatsoever to do with the evaluative truth.

Again the result would be the

implausible sceptical conclusion that our evaluative judgments are in all likelihood mostly off track^41^

This is but a sketch of a form of argument that Joyce and Street develop in great detail.^42^ It should be clear by now that the argument that Joyce and Street are putting forward is simply a more ambitious version of the very same debunking argument we've discussed earlier, only now targeting *all* of our moral or evaluative beliefs. I will set it out, as Street does, in terms of evaluative beliefs in general, not just moral ones. The first premise is therefore a causal claim about the origins of our evaluative beliefs:

*Causal premise.* Our evolutionary history explains why we have the evaluative beliefs we have.

The second premise is by now familiar claim that

*Epistemic premise.* Evolution is not a truth-tracking process with respect to evaluative truth.

which presupposes the truth of

*Metaethical assumption.* Objectivism gives the correct account of evaluative concepts and properties.

Therefore

*Evaluative scepticism*. None of our evaluative beliefs is justified.^43^

### Three variants of the global debunking argument

As I just set it out, this epistemic argument is not different in kind from the more targeted variants deployed within normative ethics. It is metaethical only in the sense that it purports to establish a truly sweeping conclusion. But Street and Joyce also believe that this global argument has further implications. And it is here that we find the most striking difference in their understanding of the argument. Street thinks it ultimately supports, not scepticism, but the truth of *anti-objectivism*; Joyce thinks it does support scepticism, but only with respect to our moral beliefs, and also that this needn't affect first-order morality. Let me offer a brief diagnosis of this disagreement.

Joyce holds, on independent semantic grounds, that objectivism *is* the correct account of moral discourse, and he therefore thinks that the argument really does establish moral scepticism. But, following Mackie's precedent, he also thinks that we have *pragmatic* reasons to hold on to our unjustified moral beliefs—to hold on to them as useful fictions:

The question of what we ought to *do*, once we have come to see that our moral discourse is a philosophically indefensible illusion, is a practical question. A neglected answer is that the discourse may be maintained, accepted, but not believed – that it may have the role of a fiction.^44^

Joyce thinks that the debunking argument can be contained in this way because he accepts an objectivist semantics *only* with respect to moral propositions: he thinks that only moral propositions make claims about ‘categorical’, attitude-independent norms. And he argues that this leaves us with *subjective* reasons to switch from an objective to a fictionalist (or for that matter subjective) understanding of morality, allowing us to go on with first-order moralizing exactly as before.

By contrast, objectivism is Street's *target*. Indeed her target is the more ambitious (and increasingly influential) objectivist view that *all* evaluative discourse (both moral and non-moral) should be understood in objectivist terms.^45^ She therefore takes the conjunction of objectivism and the evolutionary argument to imply not only moral scepticism, but total evaluative scepticism. But whereas Joyce understands objectivism to be a semantic claim, Street holds that objectivism is itself a substantive evaluative claim and as such needs to cohere with our others evaluative beliefs.^46^ This is why she can claim that the sceptical result is implausible, and that this implausible implication of objectivism is a reason to reject it.^47^

This disagreement is rooted in independent metaethical issues about the nature and scope of the objectivism/anti-objectivism divide. If we disagreed with Joyce and Street on these issues, we will end up with a further version of the argument. If we held, with Joyce, that objectivism is a true semantic claim, and that as such its truth is independent of claims about our evolutionary origins and their epistemic implications, then Street's direct route to anti-objectivism would be blocked. And if we further held, contra Joyce but with the metaethicists who are Street's target, that this semantic claim holds not only with respect to moral discourse but with respect to all evaluative discourse—including claims about our practical reasons—then Joyce's pragmatic route to fictionalism would also be blocked. For if total evaluative scepticism holds, then we could not justifiably believe that we have pragmatic reasons to adopt a fictionalist understanding of morality—we could not justifiably believe we have reasons to do *anything*. So on this understanding the global argument has a bleaker outcome: it would leave us with a debilitating global evaluative scepticism.

This third version of the global EDA would partly vindicate the worries about evolution I mentioned at the very beginning, the worries typically expressed in the form of the discredited metaphysical argument. That argument moved from a claim about the evolutionary origins of morality to *anti-objectivism* to evaluative *nihilism*. This version of the global EDA is an epistemic argument that moves from the *conjunction* of a claim about the evolutionary origins of morality *and objectivism* to evaluative *scepticism*.

For our purposes, however, this metaethical disagreement is immaterial. Although these variants of the global EDA arrive at different ultimate destinations, in one respect the end result remains the same: none leaves room for the targeted use of EDAs within normative ethics.^48^

## IV Can We Resist the Global Debunking Argument?

I think that the global EDA has considerable force. Not everyone will be equally impressed, but would-be debunkers in normative ethics certainly can't ignore it. Until this argument has been defused, they cannot deploy local EDAs in normative ethics with a clear conscience. And even if the global argument can be resisted, it might be resisted in a way that leaves no space for EDAs of *any* kind.

If we also hold that the global argument establishes total evaluative scepticism, then the task of resisting it is even more urgent. Although it makes no sense to *reject* evaluative scepticism simply because it is unwelcome, we have good epistemic reason to be wary of *endorsing* evaluative scepticism without sufficient justification, given that it would lead to a massive revision of our system of beliefs. And we have good evaluative reasons to be cautious. If we endorse evaluative scepticism by mistake, we may because of this fail to respond to many genuine values. So we have strong reasons to give the argument careful scrutiny.

I only have space to briefly consider several lines of response. My main aim is not to provide such a response, but to clarify the relation between the prospects of the global argument and those of the targeted EDAs deployed in normative ethics.

One way of resisting the global argument is to show that its conclusion can't be true. One way of doing that would be to show that evaluative scepticism is incoherent—that, for example, the global argument also applies to epistemic norms, and scepticism about these would be self-stultifying. Or it might be claimed that we simply know that certain evaluative claims are true—that, for example, it's wrong to set fire to cats for fun. And it might be claimed that we know this, and thus the falsity of the global argument's conclusion, with greater certainty than we know the truth of any of its premises—though of course this impression of certainty might itself have a debunking explanation.

Even if some such response was successful, it would remain an open question which of the argument's premises we need to reject—a question that is critical for the prospects of EDAs in normative ethics. If we reject the epistemic premise by rejecting objectivism, then targeted EDAs would also immediately lose their sting. And if we held on to objectivism, it seems to me doubtful that a plausible story can be told on which the evolutionary pressures that shaped our evaluative dispositions were at least partly tracking independent evaluative truths.^49^ And I take it that few would be attracted by the option of rejecting the theory of evolution as now understood in favour of some variant of teleological supernaturalism.^50^ In any case, the epistemic premise is shared by both the global argument and targeted EDAs and without it both would be disarmed.

This leaves us with the causal premise. The examples of targeted EDAs we considered earlier made causal claims about particular evaluative beliefs. But the causal premise of the global argument makes a sweeping claim about *all* evaluative beliefs, and it is natural to doubt whether Joyce and Street say enough to support this grand causal claim.

It might be replied that although we are very far from being able to construct an EDA for each of our evaluative beliefs, we might still have strong reason to believe that our evaluative beliefs as a whole are, as Street puts it, “thoroughly saturated with evolutionary influence.” But this claim is ambiguous. Does it refer to all or most or even just many of our evaluative beliefs? But if it isn't a claim about all of them, how does it support the sceptical conclusion?^51^ The claim might be, not just that many of our beliefs are saturated with evolutionary influence, but also that we do not know *which*, and that this means that we can no longer trust *any*. This claim, however, would by itself support only a temporary suspension of belief. It might still be possible to identify criteria for distinguishing infected and non-infected beliefs, leading us away from global scepticism to scepticism focused only on some sectors of evaluative belief—that is, back to seeing EDAs as operating locally within normative ethics. And it seems we already possess such criteria at least in outline—criteria already in use not only by evolutionary psychologists and by debunkers in normative ethics, but by Street herself, when she supports her case by pointing to examples of a range of evaluative beliefs that seem especially amenable to evolutionary explanation. Indeed the diversity of evaluative beliefs over time and across and within cultures—a diversity not fully explained by differences in non-evaluative belief—makes the suggestion that all evaluative beliefs can be given a straightforward evolutionary explanation extremely implausible. But if only some of our evaluative beliefs are susceptible to the relevant kind of evolutionary explanation, and we can at least roughly gauge the degree of this evolutionary influence on various beliefs, then what we should get isn't evaluative scepticism but a proportional lowering of justification.^52^

It might be claimed in reply that the global argument doesn't require that all of our evaluative beliefs have a straightforward evolutionary explanation, a claim that is clearly false, but only that the off track evolutionary influence on our evaluative beliefs is such that whatever further causal story needs to be told to explain why we now have the evaluative beliefs we have—a story that will presumably refer to culture, history or even practical reflection—that further story would do nothing to salvage the epistemic standing of these beliefs. So the global argument needs to be supplemented. It also needs to be supplemented because of a distinction mentioned earlier: it's one thing to claim that none of our evaluative beliefs is justified, another to claim that none *can* be justified, and yet another to claim that we are unable to justify *any* evaluative proposition, and without this last claim the resulting evaluative scepticism may only be fleeting.

There are certainly gaps in the ambitious causal story that Joyce and Street need to tell to establish the causal premise, and it remains to be seen whether the global argument can be completed.^53^ Would-be debunkers in normative ethics need to hope that this argument can't be completed, that only *some* of our evaluative beliefs are vulnerable to evolutionary debunking. But even if the global argument can be resisted in this way, we need to be realistic about what the outcome is likely to be. I think my earlier point against the current use of EDAs in normative ethics would still stand: it greatly underestimates the extent to which evaluative belief is infected by evolutionary influence.

In particular, I want to question the assumption, shared by several would-be debunkers, that once we purge our evaluative beliefs of the distortions of our evolutionary history, what will emerge is the truth of utilitarianism. Peter Singer, for example, thinks that belief in utilitarianism immune to EDAs utilitarianism because it's based on a “rational intuition” that is not “the outcome of our evolutionary past,”^54^ and that this is shown by the fact that although there may be evolutionary explanations of dispositions to reciprocal and kin altruism, the impartial form of altruism advocated by utilitarians is not adaptive and therefore not susceptible to such explanation. Even if this claim is correct, it would achieve little. If a disposition to partial altruism was itself selected by evolution, then the epistemic status of its reasoned extension should also be suspect. To see this, imagine a person who is strongly motivated to spend all of his time counting the blades of grass in his backyard. Someone goes to him and complains that he is drawing an irrelevant distinction. There is nothing special about the blades of grass in *his* backyard. If he should count anything at all, he should count *all* blades of grass in the world. But if it is pointless to count blades of grass, then it's pointless to count all blades of grass. If any EDA is successful, an EDA of partial altruistic concern must be. But this means that extending this concern through reasoning does nothing to salvage its epistemic status.^55^ Worse, utilitarianism is empty of content unless supplemented by an account of well-being. But many of our evaluative beliefs about well-being, including the beliefs that pleasure is good and pain is bad,^56^ are some of the most obvious candidates for evolutionary debunking.

Why should we expect that, if some evaluative beliefs can survive the doxastic purge, the resulting normative view would resemble any of the present competing alternatives? After all, all of these, including utilitarianism, were developed by reflection on a set of evaluative beliefs and intuitions that is at least partly infected by distorting influence. I don't know what moral theory we would get if we seriously attempt a purge of all evolutionary influences on our evaluative beliefs. But if anything would survive, it is likely to be far more counterintuitive than anything dreamed of by utilitarians. Perhaps we would need to reject the very normativity of well-being, or at least replace our current attitudes to pleasure, pain, health and death with an especially elevated form of perfectionism. These are only speculations, but, worrisomely, the view that emerges in outline is more Nietzsche than Singer.

## V Conclusion

So we are left with three possibilities. The first is that *none* of our evaluative beliefs is undermined by EDAs, meaning that EDAs have no use in both normative ethics and metaethics. This possibility would be true if none of our evaluative beliefs can be explained in evolutionary terms, or if the evolution of our evaluative capacities and sensibilities can be shown to track the evaluative truth, neither of which seems to me plausible. This outcome would also follow if some anti-objectivist view is the correct account of evaluative discourse.

The second possibility is that *all* of our evaluative beliefs are undermined by EDAs. This would be true if the global argument was successful and supported not anti-objectivism but evaluative scepticism or even just moral scepticism, meaning, again, that there is no space to use EDAs in normative ethics. This result would follow even if we believed, with Joyce, that in response we should revise our evaluative discourse to make it immune to EDAs.

The third possibility is that only *some* of our evaluative beliefs are undermined by EDAs. This is what would-be debunkers in normative ethics must assume. Given that it's not yet clear whether targeted EDAs can be prevented from collapsing into the sceptical argument, this is a precarious assumption to make at this stage. But worse, ‘some’ is likely to mean ‘very many’, including some of our most fundamental evaluative and moral beliefs. What seems utterly implausible is the possibility that EDAs can continue to have a legitimate piecemeal use in normative debate. If EDAs work at all then, in one way or another, they are bound to lead to a truly radical upheaval in our evaluative beliefs.^57^

## Notes

1 O*bjectivist* views I take to claim that evaluative propositions have truth conditions that are not grounded in our evaluative attitudes; *anti-objectivist* views (which include a range of subjectivist, response-dependent and intersubjectivist views) deny this. To save words, I will use ‘evaluative’ to refer not only to claims about value but also to claims about moral and other practical reasons.

2 Dworkin, 1996, 123.

3 Nagel, 1997 is a book length attack on this and related tendencies.

4 Hawkes, 2007.

5 Something is an undermining defeater for a belief if and only if it gives us reason to question the grounds of that belief—for example, a reason to question the reliability of the source of those grounds. See Pollock and Cruz, 1991, 196–97. I will understand debunking arguments to issue defeaters that only undermine *justification*, not defeaters that rebut *truth*. Notice that, for my purpose, it's unimportant what exact form of truth-tracking or reliability is needed to confer *positive* justification. We can tell that various processes are ‘off track’ in the relevant sense without having to endorse any specific positive epistemic account.

6 To simplify presentation, I ignore the point made above that on some views of justification the conclusion would follow only if S believed these two premises.

7 Crisp, 2006, 89.

8 For an interpretation of Marx and Nietzsche along such lines, see Leiter, 2004. By citing Gettier examples to show the relevance of aetiology to epistemic standing, Leiter gives the impression that the issue is about knowledge where it is, I think, firstly about justification.

9 Nietzsche, 1881/1982, s. 95. For more on Nietzsche's use of debunking arguments, see Reginster, 2006, 40–43.

10 Nietzsche, 1886/1986, I, 133. For an interpretation of Hume as making a similar debunking argument against fideist theism, see Kail, 2007.

11 Nietzsche, 1887/1967. Nietzsche's genealogical speculations were actually a *reaction* to Darwinian accounts of the origins of morality. A caveat: Nietzsche's metaethical views are notoriously hard to pin down, and it is also possible to interpret his critique of morality as involving a more sweeping argument that would parallel the metaethical evolutionary argument I shall be discussing below.

12 Singer, 2005, 345. See also Singer, 1993, 145–7.

13 Mulgan, 2006, 2.

14 Crisp, 2006, 121–122.

15 Reid, 1753/2000, 441.

16 This might be too quick. Even if we knew that God exists and is omnibenevolent, so long as we have no independent way of ascertaining *what* is morally right and good, we wouldn't be able to conclude that our natural dispositions track the truth. For all we know God might be a utilitarian, and disposing us to form mistaken beliefs might be the best way to promote overall happiness.

17 Sidgwick, 1879, 109–111.

18 Huemer, 2005, 219; see also Huemer, 2008.

19 Parfit, 1986, 186.

20 Crisp, 2006, ch. 3.

21 Rachels and Alter, 2005, 314–315. Rachels discusses ‘aetiological’ grounds for criticising evaluative claims in his 2003.

22 Greene, 2008.

23 Singer, 2005, 348. Some of Singer's remarks suggest that he may not distinguish this argument from one that relies on the premise that evolution *is* truth tracking, but only relative to the circumstances holding in the ancestral environment. Such arguments wouldn't directly undermine an evaluative belief but rather establish that it's a ‘moral heuristic’, a rule of thumb that applies in certain circumstances but not in others. This claim about evolution implies that if, say, indirect ways of killing people *were* present in the ancestral environment, evolution *would have* selected the morally correct response. (And in that case, wouldn't evolution have selected the ultra-deontological aversion *both* to pushing someone to his death to save five *and* to more indirect ways of killing?) But this claim about evolution is extremely implausible. Surely it's implausible that natural selection, guided by the Principle of Utility, inculcates such moral heuristics to promote the greater good.

24 Greene, 2008, 63.

25 Both Greene and Singer present Greene's neuroimaging findings as playing a crucial part in supporting these claims. But if their argument was that (1) brain imaging shows deontological intuitions to have their source in affective responses, and that (2) affective processes are ‘off track’, then it would be a straightforward psychological debunking argument, and reference to evolution would be redundant (though I do not think a serious case has been made in support of either of these premises). However, if deontological intuitions do have their source in ‘primitive’ brain areas associated with affect, this might provide weak evidence that they have their source in innate dispositions, and therefore weak support for the causal premise of an EDA.

26 For a response to historical debunking arguments against Christian belief, see Plantinga, 2000, ch. 11.

27 Street appears to make this assumption in Street, 2006, 112–113, and it is of course suggested by the earlier Huemer quote.

28 This shouldn't be confused, however, with the ongoing debate over whether there is an innate domain-specific morality module. The claim that some evaluative disposition is innate doesn't commit us to the existence of such a module.

29 See Street, 2006, 125–135, and Joyce, 2006a, ch. 6.

30 This assumption was famously challenged in Plantinga, 1993, ch. 12; Nagel, 1997 seems to endorse Plantinga's argument.

31 Non-naturalist evaluative realism is a paradigmatic form of such objectivism. It's more debatable, however, whether EDAs are really compatible with some naturalist forms of realism (see fn. 42)—and even whether they are *incompatible* with some sophisticated forms of noncognitivism.

32 As Nicholas Sturgeon notes, Gilbert Harman's explanation of the commonsense moral distinction between harming and failing to aid as having its origins in bargaining between the weak and the strong would count as debunking to objectivists yet is taken to have no such implications by a relativist such as Harman (Sturgeon, 1992). Notice that if anti-objectivism is incompatible with EDAs, it's also likely to be incompatible with *historical* debunking arguments. I should add that I don't mean to deny that there might be ways in which, on some conceivable anti-objectivist views, facts about causal origins *could* have negative epistemic implications. But this wouldn't show that debunking arguments in the present sense are compatible with anti-objectivism. I'm grateful here to Tamler Sommers.

33 Greene, 2008, 76.

34 Joyce, 2000, 2006a, ch. 6, 2006b. This argument was anticipated by Ruse, 1998, 252–54; Ruse and Wilson, 1986, 186–7, don't distinguish this epistemic argument from the discredited metaphysical argument mentioned at the beginning.

35 Darwin, 1871/2004, 122. For a similar thought experiment, see Ruse & Wilson, 1986, 186.

36 Joyce, 2006b, 133.

37 Joyce, 2006b, 135.

38 Joyce's argument is explicitly modelled after Gilbert Harman's causal explanatory argument against objectivist realism. But whereas realists can just deny that moral facts need to meet any such explanatory condition, it is harder for them to claim that our evaluative beliefs would be justified even if formed by off track processes.

39 Street, 2006. Street uses ‘realism’ to refer to what I call ‘objectivism’.

40 Street, 2006, 114.

41 Street 2006, 122.

42 Naturalist metaethical views that identify normative facts with natural ones introduce complications that I ignore here, and which are addressed at length by both Joyce and Street. Note that if these naturalists views turn out to be immune to the global debunking argument, then they will also rule out the targeted use of such arguments in normative ethics.

43 Might some of our evaluative beliefs still be true by luck? Since we lack a grip on the probability of this occurring that is independent of our existing evaluative beliefs, we can't say. If we *do* assume something like this set of beliefs, then it does seem extremely unlikely that a random pairing of evaluative and descriptive predicates would yield a true evaluative proposition.

44 Joyce, 2000, 730.

45 Different versions of this view have been recently defended by Derek Parfit, Thomas Scanlon, Joseph Raz, Russ Shafer-Landau and others.

46 For the view that the objectivism/anti-objectivism divide is a substantive evaluative matter, see Dworkin, 1996.

47 Street, 2006, 141. However, if we took the falsity of evaluative scepticism as a truism that any plausible metaethical views must preserve, then the global argument could still be used against objectivism even if objectivism is understood as a metaethical view.

48 A fourth possible variant would accept Joyce's view about the scope of objectivism but deny that we (or at least many of us) have pragmatic reasons to hold on to morality. This would leave us only with the reasons generated by our subjective nonmoral concerns. This fourth destination is obviously also incompatible with the targeted use of EDAs.

49 Though see Huemer, 2005 and Enoch, 201.

50 As Nagel seems to when he writes that “if evolutionary naturalism is the whole story about what we take to be practical reasoning, then there really is no such thing,” and concludes that naturalism must be supplemented by some teleological principle (Nagel, 1997, ch. 6).

51 It might be replied that Street, at least, may not need the argument to support evaluative scepticism. Isn't it enough if it can show that *many* of our evaluative beliefs are in error—wouldn't that be, by her lights, a sufficiently implausible implication of objectivism? But on at least one understanding of ‘many’, a utilitarian such a Singer would be entirely happy to endorse this implication.

52 See also Huemer, 2008.

53 In Street, for example, we find the suggestion that practical reflection must necessarily rely on some prior range of evaluative commitments, and that therefore reflection can't put us back on track given that the initial range of human concerns *is* susceptible to straightforward evolutionary explanation (Street, 2006, 123–125)—claims that objectivists are likely to reject. And Joyce doesn't always distinguish the sceptical argument from parallel arguments about the evolutionary origins of our evaluative *concepts* or of the *‘moral sense*’ (see e.g. 2006b, 135). A debunking argument that successfully targeted these would certainly address the problem, but, in showing we are unjustified in believing objective values *exist*, it might establish not scepticism but nihilism. For extensive criticism of the causal premise, see Parfit, forthcoming.

54 Singer, 2005, 350–351. Heumer also thinks the odds favour utilitarianism (2008, 390).

55 Huemer thinks we can resist the sceptical argument by appealing to the positive evidential force of coherence (Huemer, 2008, pp. 379–380). But the coherence of clusters of evaluative belief might itself be due to evolutionary pressures. Although it would be an exaggeration to say that our evaluative dispositions face the tribunal of evolution not one by one but as a whole, adaptive pressures would work in obvious ways to favour dispositions to form evaluative beliefs that are mutually coherent.

56 Rachels and Alter claim that our evaluative beliefs about pain are immune to debunking because they are formed in a way akin to direct perception (Rachels and Alter, 2005, 315), and Nagel thinks that to believe a debunking evolutionary explanation of pain's badness would be insane (Nagel, 1989, 157). But as Street argues, there *is* an obvious evolutionary explanation of our belief that pain is bad, an explanation that needn't cite any claim about the truth of this conviction (Street, 2006, 144ff.). I've developed a similar argument in my unpublished doctoral dissertation.

57 Earlier versions of this paper were presented at the University of Oslo, the Hong Kong City University, where my respondent was Joseph Lau, and at Princeton University, where my respondent was Gilbert Harman. I am grateful to them, and to the audiences in these talks, for extremely useful comments. I also greatly benefitted from comments by Derek Parfit and an anonymous referee, and from suggestions by S. Matthew Liao, Ingmar Persson and James Morauta. This work was supported by the Wellcome Trust [WT087208MF].
